# Molecular characteristics of *Neisseria meningitidis* in Qatar

**DOI:** 10.1038/s41598-021-84262-1

**Published:** 2021-02-26

**Authors:** Manal Mahmoud Hamed, Fayaz Ahmad Mir, Emad Bashier Ibrahim Elmagboul, Abdullatif Al-Khal, Muna A. Rahman S. Al. Maslamani, Anand Sarwottam Deshmukh, Hamad Eid Al-Romaihi, Mohd. Ahmed M. Sharif Janahi, Fatma Ben Abid, Adila Shaukat Ali Kashaf, Gulab Sher, Vinod Kumar Gupta, Godwin J. Wilson, Junais Kadalayi, Sanjay H. Doiphode

**Affiliations:** 1 Hamad Medical Corporation, Doha, Qatar; 2 Ministry of Health, Doha, Qatar

**Keywords:** Pathogens, Molecular medicine, Bacterial infection, Meningitis

## Abstract

The aim of the current study is to review the molecular characteristics of *Neisseria meningitidis *(*N. meningitidis*) in Hamad Medical Corporation, which is the provider of secondary and tertiary care in the state of Qatar. A total of 39 isolates of *N. meningitidis* from the period of 2013 to 2018 were revived and identified by Vitek, and susceptibility on the basis of the E test was retrieved from the patient’s files. The revived isolates were subjected to multilocus sequence typing. The most common serogroup (19) of *N. meningitidis* was W135, of which 12 were isolated from blood and CSF. ST-11 was the most predominant ST clonal complex causing *N. meningitidis* cases (61.53%). Clonal complex ST-41/44 was the second most observed complex (3, 2 of which were related to serogroup B). The most frequent sequence type was 9596 (8 isolates). Determining the molecular pattern of *N. meningitidis* in Qatar is helpful for understanding the strains circulating in Qatar, and the study of the resistance trend of such strains may be very helpful for empirical treatment of future patients.

## Introduction

Invasive *Neisseria meningitidis *(*N. meningitidis*) infection (meningitis, meningococcemia) is associated with high mortality and morbidity worldwide despite the use of proper antibiotics. Fever, bacteremic pneumonia, pericarditis, septic arthritis, urethritis and ear infection are less frequent presentations^1,2^.

Capsules of *N. meningitidis* are important virulent factor that allow the organisms to escape human immunological defense mechanisms. The organism is known to change its capsular polysaccharide by capsular switching to evade the immune system; thus, the introduction of vaccine serotypes might result in an increase in serotypes that are not included in the vaccines by a mechanism such as capsular switching. Genetic changes have also been observed after the introduction of an outer protein-based vaccine for serogroup B, with an increase in serotypes C and Y alongside major changes in outer membrane proteins^3^. The genetic makeup changes are due to either the acquisition of foreign DNA from other strains and genera or other mechanisms, such as the recombination of self-genes. Capsular switching results in a change in the serogroup of the *N. meningitidis* strain due to a change in capsular polysaccharides but will reserve the same lineage, as shown by MLST. Capsular switching was proven during the Hajj season in the Saudi Arabia outbreak caused by serogroup W-135^3^.

*N. meningitidis* can be classified by capsular polysaccharides into twelve serogroups (previously thirteen serogroups, but group D is no longer considered a separate group). Among the twelve capsular polysaccharide serogroups, only five (A, B, C, W-135, and Y) are known to frequently lead to the development of disease worldwide. Most *N. meningitidis* found in the throat are untypable or lack a capsule that is required for invasive infection. Serogroup distribution differs in different parts of the world; serogroup A is the most commonly isolated serogroup in sub-Saharan Africa^4,5^, while serogroups B, C, and Y are the most common serogroups found in Europe and the United States (US). In Asia, serogroup A is the predominant serogroup^5^. Most cases in the US are sporadic, but outbreaks are nonetheless a common occurrence. Epidemics are known to occur in sub-Saharan Africa (in 25 countries), with the most recent outbreak occurring during the Hajj season in Saudi Arabia and caused by serogroup W-135^6,7^.

A recent method of characterization of *N. meningitidis* is molecular typing methods, which are used to study the relatedness of outbreak isolates by detecting minimal changes in the genome. Molecular typing tests detect variations to characterize the clones circulating in a certain geographical area and their closeness to any of the known international clones. An example of these tests is MLST, which has become the most widely used test worldwide^6–8^.

MLST, the gold standard for molecular typing, classifies meningococcal strains into different sequence types (STs) based upon polymorphisms in seven housekeeping genes. This technique helps identify any hyperinvasive lineages, i.e., meningococcal STs that are independent of serogroups and are disproportionately associated with disease relative to carriage levels. Seven-locus MLST has been recommended at least for a representative selection of each country or for enhanced regional surveillance, and this information is essential for the national and international management of meningococcal disease and the study of meningococcal population biology and evolution^7,8^.

In a 2-year study conducted in Qatar (1998–2000) the leading causes of meningitis were revealed to be *Streptococcus pneumonia*, which resulted in 30% of the cases of meningitis included in the study, *Haemophilus influenzae* (24%), and *N. meningitidis* (5%); however, these findings may not reveal the actual numbers, as partially treated cases were excluded from the study. The rather low number caused by *N. meningitidis* was explained at that time by the adequate number of people who opted to receive the meningococcal vaccine in Qatar before they left for Hajj or Umrah, as requested by the Kingdom of Saudi Arabia^9^.

Resistance to antibiotics, especially penicillin, has been reported worldwide due to mutations in the constitution of penicillin-binding protein, PBP 2, and less commonly due to the production of penicillinases. The occurrence of penicillin resistance in the closely related *Neisseria gonorrhea* drove researchers to also consider the possibility of developing resistance in *N. meningitidis*. Commonly used antibiotics are third-generation cephalosporins, such as ceftriaxone. Resistance to rifampin and trimethoprim/sulfamethoxazole is rare, but reports of resistance have been published^1,10–12^.

Identification of the major serogroups will help in understanding the efficacy of vaccine coverage and the main circulating serogroup in Qatar. Genetic characterization is essential in understanding the characteristics and magnitude of circulating clones and provides national authorities in Qatar with valuable data about the efficacy of the vaccine policy and vaccination schedule and the effectiveness of the available vaccine as well as suggestions for strategies for better control of *N. meningitidis* infections and spread^13^.

To the best of our knowledge, there has been no study of the genetic characteristics of *N. meningitidis* in Qatar or any analysis comparing the Qatari invasive strains with international strains. This study represents the first report of *N. meningitidis* molecular characteristics in Qatar.

## Methods

We subjected our strains to molecular typing techniques for epidemiological purposes to investigate potential outbreak strains in Qatar. All strains that were stored in our repository (in the period between February 2013 and March 2018) were revived and subjected to the following procedures:

### Isolation and culture

All meningococcal isolates, principally invasive isolates in Qatar representing all known invasive genotypes and serogroups, stored in our cryorepository were tested.

The lyophilized *N. meningitidis* cryovials were removed from the Hamad General Hospital repositories, where they were preserved at − 80 °C, and allowed to thaw for 10 min in a biological safety cabinet. The tubes were then opened, and beads from each cryovial were transferred to chocolate agar plates by using a sterile needle. The chocolate agar was streaked for bacterial isolation, and the beads were discarded in a yellow sharps box. The streaked chocolate agar plates were sealed with parafilm and incubated at 37 °C in a microaerophilic incubator with a 5–10% CO2 supply for 18 h. After an 18-h incubation period, smooth, moist, glistening, and convex colonies of *N. meningitidis* had grown. The colonies were subcultured onto a fresh chocolate agar plate and incubated at 37 °C in a microaerophilic incubator with a 5–10% CO2 supply for another 18 h. The next day, the *N. meningitidis* colonies were subjected to DNA extraction.

Antimicrobial susceptibility, serogrouping and all patient details were retrieved from the patient files. The serogrouping used antisera against the major serogroups (A, B, C, W-135, X, and Y), which cause life-threatening disease^14,15^.

### DNA extraction and sequencing

DNA was extracted by the PureLink Genomic DNA Mini Kit (Invitrogen) according to the manufacturer’s instructions. In brief, *N. meningitidis* colonies were transferred to 180 μL Digestion Buffer, 20 μL of Proteinase K was added, and the tube was briefly vortexed. The tube was incubated at 55 °C with occasional vortexing until lysis was complete. Then, 20 μL of RNase A was added to the lysate, mixed well by vortexing and incubated at room temperature for 2 min. Two hundred microliters of lysis/binding buffer was added to the lysate and mixed well by vortexing to obtain a homogeneous solution. Then, 200 μL of 96–100% ethanol was added to the lysate and mixed well by vortexing for 5 s to obtain a homogeneous solution. The lysate (~ 640 μL) was added to the spin column and centrifuged at 10,000 × *g* for 1 min. The collection tube was discarded, and the spin column was transferred into a clean collection tube. Then, 500 μL of wash buffer 1 was added to the column and centrifuged at 10,000 × *g* for 1 min. The collection tube was discarded, the spin column was transferred into a clean collection tube, and 500 μL wash buffer 2 was added to the column and centrifuged at maximum speed for 3 min. The collection tube was discarded, and the spin column was transferred to a sterile 1.5-mL microcentrifuge tube. Then, 50 μL of elution buffer was added to the column, incubated at room temperature for 1 min and centrifuged at maximum speed for 1 min. The eluted DNA was stored at − 20 °C. All centrifugations were carried out at room temperature^8,16^.

### Multilocus sequence typing (MLST) and FetA typing

MLST on patient isolates was performed in HMC laboratories, and MLST and FetA typing were performed as previously described using the primers listed on the PubMLST website (http://pubmlst.org/neisseria/)^8,16^. PCR was carried out using Maxima Hot Start Master Mix (Thermo Scientific) in 25 μL of reaction with 12.5 μL Master Mix (2X), 1 μL (10 μM) forward primer, 1 μL (10 μM) reverse primer, 2 μL DNA template, and 8.5 μL nuclease-free water. The thermal cycling conditions were as follows: initial denaturation at 94 °C for 2 min; 35 cycles of 94 °C for 1 min, 55 °C for 1 min, and 72 °C for 1 min; and a final extension at 72 °C for 2 min. The amplification was checked by running 5 µl of each reaction product on a 2% agarose gel with a 100 bp DNA ladder (Invitrogen). PCR products were purified by ExoSAP-IT reagent (USB, Affilmetrix). In brief, 5 μL of PCR product was mixed with 2 μL of ExoSAP-IT reagent and incubated at 37 °C for 15 min, followed by 80 °C for 15 min. Sequencing was carried out by the BigDye Terminator ver. 3.1 cycle sequencing kit (Applied Biosystems) in a 5 μL reaction volume consisting of 2 µL of BigDye Terminator 3.1 Ready Reaction Mix, 2 µL of forward/reverse primer (0.6 µM), and 1 µL of purified PCR product. The thermal cycling conditions were 25 cycles of 96 °C for 10 s, 50 °C for 5 s, and 60 °C for 2 min. After completion of PCR, 15 μL of sterile dH2O was added to each sample to a final volume of 20 μL. Sequencing reaction products were purified by a BigDye XTerminator purification kit (Applied Biosystems) according to the manufacturer’s instructions. Then, 90 µL of SAM solution was mixed with 20 µL of BigDye XTerminator bead solution to prepare the SAM/BigDye XTerminator bead working solution, which was added to each sample. Then, the sample was mixed on a vortexer for 30 min and centrifuged at 1000 × *g* for 2 min. The purified sequencing reaction products were analyzed on an ABI 3500 Genetic Analyzer (Applied Biosystems) according to the manufacturer’s instructions. Designation of sequence types (ST) and clonal complexes (CC) were retrieved by comparison on the PubMLST website (http://pubmlst.org/neisseria/)^8,16^.

### Ethical approval

I confirm that all methods were carried out in accordance with the relevant guidelines and regulations. I received approval for this research from Qatar Medical Research Center and obtained an exemption from Hamad Medical Corporation IRB regarding consent, as this study dealt with retrospective bacterial isolates stored in our repositories and not patients. The reference code is Research Proposal #15453/15. I declare that my research in Qatar was conducted ethically and in accordance with community expectations.

## Results

**Figure 1:**
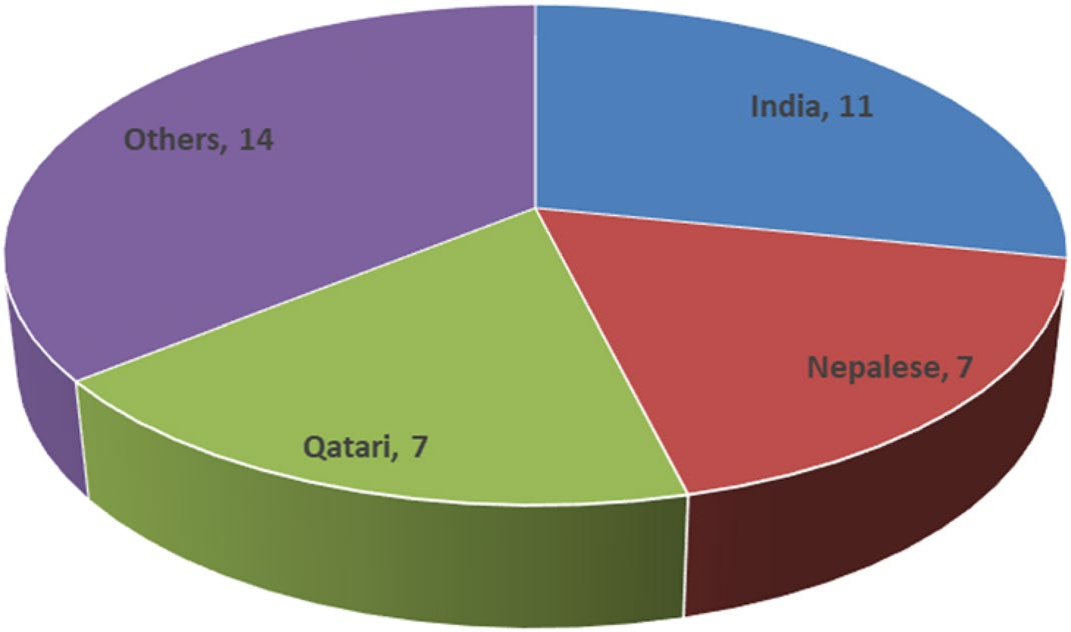
Nationality distribution among the patients.

Thirty-nine isolates of *N. meningitidis* were detected from February 2013 to March 2018, of which 35 were from Hamad General Hospital (HGH) and four were from Al Wakra Hospital. The patients were predominantly of Indian nationality (11) (Fig. 1).

Twenty-three patients presented with fever, of whom 12 presented with additional meningitic symptoms and signs (headache, rash, irritability). Six patients were referred from the medical commission due to abnormal chest X-ray. Five out of the six had pulmonary tuberculosis, and all six *N. meningitidis* isolates were isolated from respiratory tract samples (two were serogroup W135, two serogroup A and two untypable) (Table S1, supplementary file).

**Figure 2:**
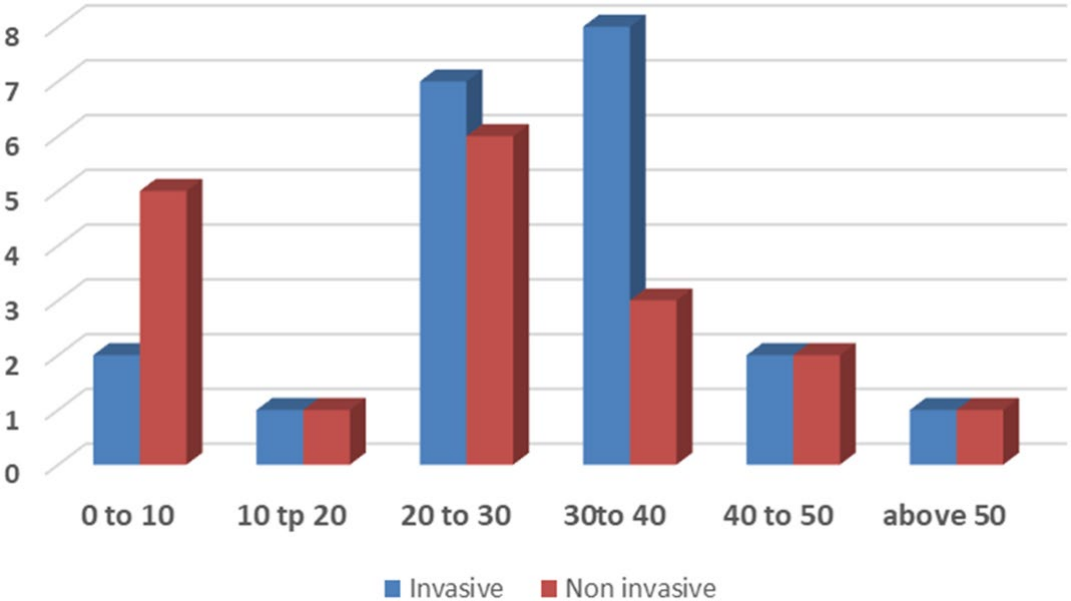
Age distribution among patients.

Of the patients, 15 (38%) were aged 20–30. There were more invasive isolates in the 30–40 age group than in all the other age groups (Fig. 2).

**Figure 3:**
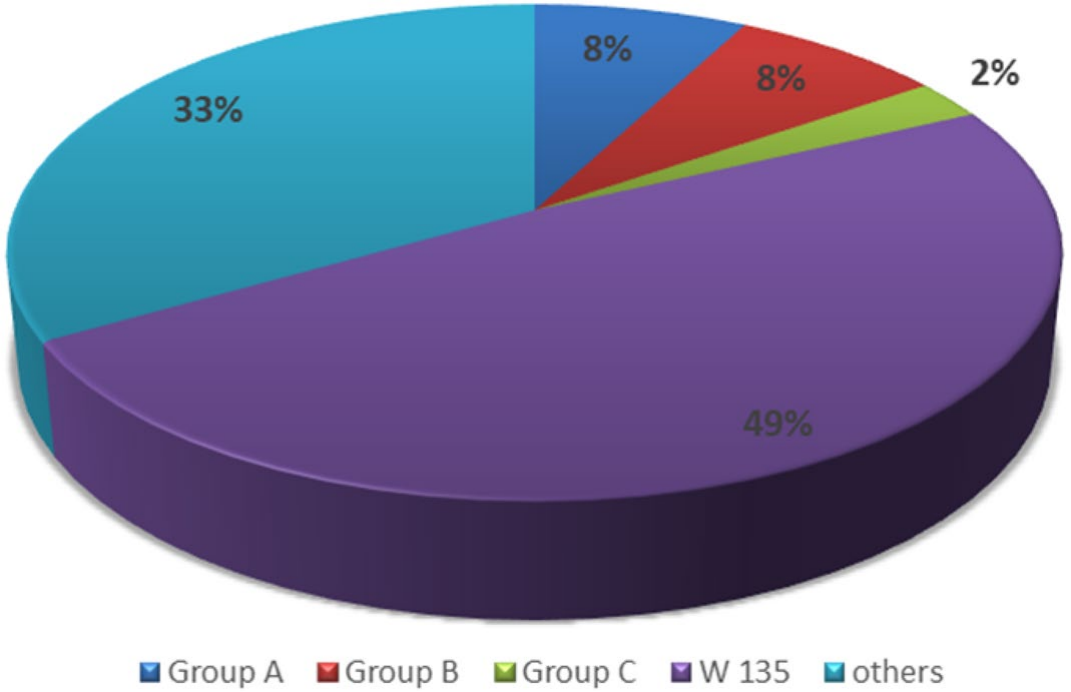
Serogroup distribution among all isolates.

**Figure 4:**
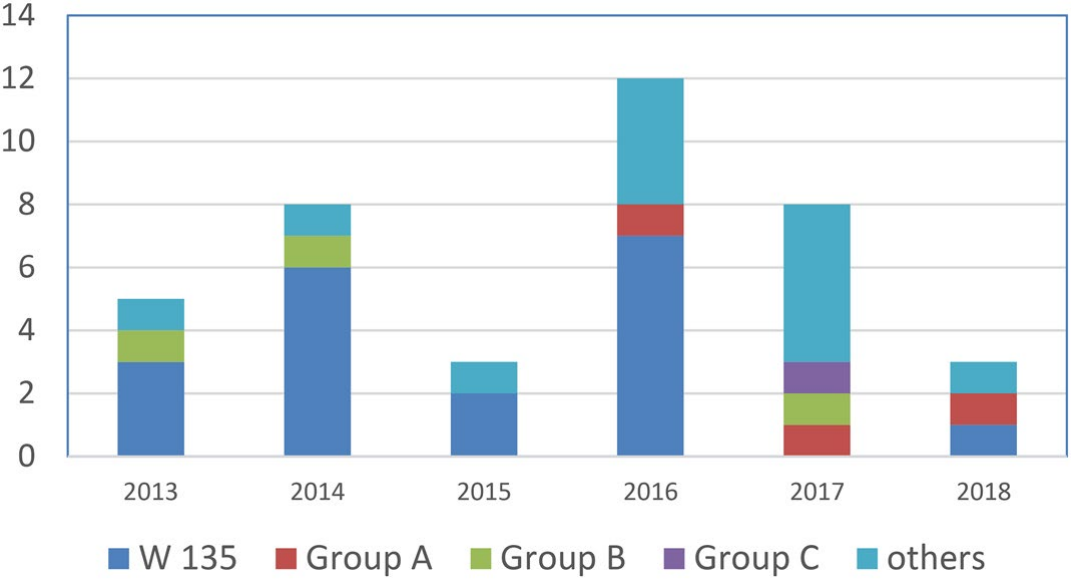
Occurrence of *Neisseria meningitidis* serotypes.

Twenty-one isolates were recovered from blood specimens, one from CSF (56% invasive disease), and 18 from the respiratory tract. The most common serogroup (19) of *N. meningitidis* was W135 (49%), of which 12 were isolated from blood and CSF, followed by group B (3) and group A (3). One isolate was related to group C, and nine *N. meningitidis* isolates were of unknown or untypable groups (Fig. 3). The maximum number of cases occurred during 2016 (12 cases, mainly Nepalese and Indian patients, with 10 of the 12 isolated from respiratory samples) (Fig. 4).

Penicillin susceptibility appeared in only 61%, while ciprofloxacin susceptibility was 89% and meropenem and ceftriaxone susceptibility was 100% (Table 1).

**Table 1:** Antimicrobial susceptibility testing for *Neisseria meningitidis*.

Antibiotics	MIC (µg/mL) breakpoints interpretation			Antimicrobial susceptibility (%)		
	S	I	R	S	I	R
Penicillin	≤ 0.006	0.12–0.25	≥ 0.5	61	32	8
Ceftriaxone	≤ 0.12	–	–	100	–	–
Ciprofloxacin	≤ 0.03	0.06	≥ 0.12	89	5	5
Meropenem	≤ 0.25	–	–	100	–	–
Rifampin	≤ 0.5	1	≥ 2	85	12	3

Out of the 39 isolates, 24 isolates had clonal complex ST-11 (61.5%), of which 18 isolates had serogroup W 135 (75%). Four isolates were found that were not related to any clonal complexes in PubMLST. We anticipate that these isolates must be related to novel complex(es) and thus need further evaluation. The serogroups of these four isolates were groups A, B, and W135, and one was untypable. Clonal complex ST-41/44 was the second most observed complex (three, two of which were related to serogroup B). The most frequent sequence type was 9596 (eight isolates), followed by 7979 (seven), all of which belonged to clonal complex ST-11 (Table S1, supplementary file).

## Discussion

HMC is the main governmental hospital serving the whole state of Qatar and providing every level of care to residents of the country. Therefore, all cases of suspected meningitis are treated at HMC. We isolated a total of 39 isolates of *N. meningitidis* that were detected by culture from 2013 to March 2018 (Table S1, supplementary file). More than half of these isolates caused invasive diseases (meningitis and/or bacteremia), and 12 cases occurred in 2016 (Fig. 3), of which seven were related to serogroup W 135(58%) and three were invasive isolates (Table S1, supplementary file). The number of cases per year varies from a minimum of 3 cases in 2015 to a maximum of 12 in 2016 (Fig. 3). The lack of data from the Middle East, especially the Gulf Cooperation Council (GCC) countries, provided us with an opportunity to fill that gap with this study; thus, we aimed to review the molecular epidemiology of *N. meningitis* in Qatar.

The most common serogroup (19) of *N. meningitidis* isolated at HMC was W135 (49%), which is similar to the *N. meningitidis* outbreak that occurred during pilgrimage to Saudi Arabia in 2000, with W135 (37%) being the main serogroup, followed by group A (24%). Many countries became involved as the patients returned to their home countries. An earlier outbreak with serogroup A occurred in Middle East and North African countries, including Qatar, in 1988^5^.

The predominant serogroups vary with the region. The main serogroup in sub-Saharan Africa was group A, but since the introduction of a vaccine for group A, outbreaks caused by this serogroup have started to disappear, and serogroups X and W135 have started to increase, while in the USA, serogroups B, C, and Y are the main causes of meningitis. Serogroup B is the main serogroup causing meningitis in Europe, followed by W135 and C^5,17^.

Serogroup B vaccines have also been used to control outbreaks in the Saguenay Lac-Saint-Jean region in Canada and other outbreaks at US universities^18,19^.

In 2015, national immunization programs introduced Serogroup B vaccines to infants in the UK and Ireland, and regional programs were introduced in Italy^20^.

In our study, 9 serogroups were untypable, and further research is required to assess the exact serogroups of these isolates.

In Qatar, the currently available vaccine for meningococcal infections is the quadrivalent conjugated meningococcal vaccine for serogroup ACYW135, which has been widely introduced in many countries.

Our main age group consisted of persons between 20 and 40 years old. Since most of our patients were laborers and expatriates, it will be highly prudent to develop labor surveillance studies for carriage and vaccination among this group if a high carrier rate is detected among them (Fig. 2).

This is similar to what is happening in the USA and Europe, where people aged 16 through 23 and those less than 1 year old have the highest rates of meningococcal disease^19^. In our study, 2 isolates out of 39 were from children aged < 10 years, which may suggest the possibility of underestimation of the invasive disease burden caused by *N. meningitidis* in children due to early intake of antibiotics. Therefore, meningococcal disease surveillance programs are strongly recommended to determine the carrier state within this group, which may provide a clear view of the circulation of the organism within this population and may encourage the implementation of school-based vaccination programs if high carriage rates are found. Kim et al. reported in prospective population surveillance performed among children aged < 5 years that *N. meningitidis* was the most prevalent pathogen for invasive disease^20^.

Bogaert et al. studied the prevalence of *N. meningitidis* carriage among Dutch children aged 1–19 years and found a 1.5% incidence, especially among children aged 1 and above 15 years old^21^.

While carrier surveillance study among children up to 14 years of age in Egypt and Turkey, presented a wide range of carriage (1.2–18.8%), it was even higher among military staff and students, while in Israel, 29% of children aged from 11 to 15 years old were carriers of *N. meningitidis*^5^.

Our main ST clonal complex (24 isolates) was ST-11, of which 18 isolates had serogroup W135 (75%) (Table 1, supplementary file). This finding is similar to the findings of the 2017 report of The European Centre for Disease Prevention and Control (ECDC), which reported an increase in serogroup W 135 with a single clone, ST-11, in the UK as well as other countries in Europe, such as Italy and the Netherlands. In the meningitis belt within sub-Saharan Africa, there are 2 main hypervirulent clonal complexes, ST-5 and ST-11 (mainly the W135 serogroup), but there are fewer W135 epidemics than group A epidemics in this part of the world. A detailed guide was drafted by the WHO to hamper the rate of incidence for outbreaks within this region, including a strategy for chemoprophylaxis, vaccines, accurate laboratory diagnosis, and quality evaluation^22,23^.

Among our isolates, penicillin susceptibility was 61%, ciprofloxacin susceptibility was 89%, and meropenem and ceftriaxone susceptibility was 100%. In a 2004 *N. meningitidis* surveillance study in Croatia, 17 out of 23 isolates were serogroup B, 4 were serogroup C, 1 was serogroup W135, and 1 was untypable. All serogroup C isolates were resistant to penicillin, while all other serogroups were sensitive to all antibiotics (penicillin, ceftriaxone, chloramphenicol, and ciprofloxacin)^24^.

In a Turkish study of children (0–18 years) who had meningococcal meningitis, 8.7% of the isolates were resistant to penicillin, 22.8% were intermediately susceptible, and all isolates were susceptible to cefotaxime^25^. A Chinese study found an increase in resistance to quinolones (67.7%) among *N. meningitidis* isolates acquired from horizontal transfer from commensal Neisseriae (99.3%)^26^. In a UK study, two people who had returned from Hajj were found to have conjunctivitis caused by *N. meningitidis*, and a third person who had been in contact with one of them had invasive disease from the same untypable *N. meningitidis* isolate, which was resistant to ciprofloxacin and intermediately susceptible to penicillin^27^.

Regular continuous surveillance of *N. meningitidis* disease and stratified carriage among different populations, especially children and laborers, and regular publication are essential to determine the magnitude of the disease in a population and thereafter provide adequate intervention. Moreover, active early detection and study of circulating invasive strains, either locally or internationally, will help to prevent future outbreaks and decrease the incidence of invasive disease.

## Supplementary Information

Supplementary Table S1.
