# Supplementary material for: Molecular characteristics of Neisseria meningitidis in Qatar
Source: Sci Rep. 2021 Feb 26;11:4812. doi: 10.1038/s41598-021-84262-1 (PMC7910605; doi:10.1038/s41598-021-84262-1)
Supplement: Supplementary file 1 — Supplementary Table S1. [file 41598_2021_84262_MOESM1_ESM.docx]

| **Sample** | **collection** | **Gender** | **Age** | **Serogroups** | **Clonal complex** | **Sequence type** | **FetA** |
| --- | --- | --- | --- | --- | --- | --- | --- |
| 1 | Jan-18 | Male | 23 | W 135 | ST-11 | 9596 | F1-121 |
| 2 | Jan-18 | Male | 39 | W 135 | ST-11 | 13255 | F1-1 |
| 3 | Jan-18 | Male | 33 | W 135 | ST-11 | 9596 | F1-121 |
| 4 | Jan-18 | Male | 28 | W 135 | ST-11 | 1278 | F1-203 |
| 5 | Jan-18 | Male | 31 | W 135 | ST-11 | 10805 | F1-121 |
| 6 | Jan-18 | Male | 41 | Group B | ST-41/44 | 191 | F5-80 |
| 7 | Jan-18 | Male | 36 | W 135 | ST-11 | 10805 | F1-121 |
| 8 | Jan-18 | Male | NA | others | ST-11 | 10805 | F1-203 |
| 9 | Feb-18 | Male | 27 | W 135 | ST-11 | 10805 | F1-121 |
| 10 | Feb-18 | Male | 30 | W 135 | ST-11 | 7979 | F1-121 |
| 11 | Feb-18 | Male | 23 | Group B | ST-41/44 | 2609 | F1-121 |
| 12 | Feb-18 | Male | 47 | W 135 | ST-11 | 9596 | F1-121 |
| 13 | Feb-18 | Male | 25 | W135 | ST-11 | 9596 | F1-203 |
| 14 | Feb-18 | Male | 53 | W 135 | ST-11 | 7979 | F4-64 |
| 15 | Feb-18 | Male | 37 | others | ST-23 | 23 | F1-121 |
| 16 | Feb-18 | Male | 23 | W 135 | ST-11 | 9596 | F1-121 |
| 17 | Feb-18 | Male | 7 | W 135 | NEW | 3574 | F1-121 |
| 18 | Feb-18 | Male | 39 | others | ST-11 | 7979 | F1-121 |
| 19 | Feb-18 | Male | 26 | W 135 | ST-11 | 7979 | F1-121 |
| 20 | Feb-18 | Male | 44 | W 135 | ST-11 | 7979 | F1-121 |
| 21 | Feb-18 | Male | 52 | others | ST-167 | 871 | F1-203 |
| 22 | Feb-18 | Male | 33 | Group A | NEW | 3558 | F1-203 |
| 23 | Feb-18 | Male | 20 | Group A | ST-175 | 175 | F1-121 |
| 24 | Feb-18 | Male | 11 | others | ST-175 | 157 | F1-121 |
| 25 | Feb-18 | Male | 4 | others | ST-11 | 14213 | F1-121 |
| 26 | Feb-18 | Male | 13 | others | ST-1157 | 12430 | F1-121 |
| 27 | Feb-18 | Male | 33 | Group C | ST-4821 | 6928 | F1-121 |
| 28 | Feb-18 | Female | 29 | W 135 | ST-11 | 14213 | F1-203 |
| 29 | Feb-18 | Female | 4 | W 135 | ST-11 | 6066 | F1-203 |
| 30 | Feb-18 | Female | 8 | others | NEW | 3558 | F1-121 |
| 31 | Mar-18 | Male | 45 | W 135 | ST-11 | 9596 | F1-121 |
| 32 | Mar-18 | Male | 30 | W 135 | ST-11 | 9596 | F1-121 |
| 33 | Mar-18 | Male | 39 | others | ST-11 | 7979 | F1-121 |
| 34 | Apr-18 | Female | 2 | others | ST-41/44 | 6057 | F1-121 |
| 35 | Apr-18 | Male | 10M | Group B | NEW | 5656 | F1-121 |
| 36 | Apr-18 | Male | 25 | others | ST-23 | 5544 | F1-203 |
| 37 | Apr-18 | Male | 40 | W 135 | ST-11 | 9596 | F1-121 |
| 38 | Apr-18 | Male | 25 | others | ST-175 | 356 | F1-203 |
| 39 | Apr-18 | Male | 23 | Group A | ST-11 | 7979 | F5-80 |

Table-S1: Summary of epidemiologic data of N. meningitidis isolated in Qatar in 2018. *Abbreviations: MLST, multilocus sequence typing; CC, clonal complex; NA, not available.
